# Rescue of DNA-PK Signaling and T-Cell Differentiation by Targeted Genome Editing in a *prkdc* Deficient iPSC Disease Model

**DOI:** 10.1371/journal.pgen.1005239

**Published:** 2015-05-22

**Authors:** Shamim H. Rahman, Johannes Kuehle, Christian Reimann, Tafadzwa Mlambo, Jamal Alzubi, Morgan L. Maeder, Heimo Riedel, Paul Fisch, Tobias Cantz, Cornelia Rudolph, Claudio Mussolino, J. Keith Joung, Axel Schambach, Toni Cathomen

**Affiliations:** 1 Institute for Cell and Gene Therapy, University Medical Center Freiburg, Freiburg, Germany; 2 Center for Chronic Immunodeficiency, University Medical Center Freiburg, Freiburg, Germany; 3 Institute of Experimental Hematology, Hannover Medical School, Hannover, Germany; 4 Spemann Graduate School of Biology and Medicine (SGBM), University of Freiburg, Freiburg, Germany; 5 Molecular Pathology Unit, Massachusetts General Hospital, Charlestown, Massachusetts, United States of America; 6 Department of Pathology, Harvard Medical School, Boston, Massachusetts, United States of America; 7 Department of Biochemistry and Mary Babb Randolph Cancer Center, Robert C. Byrd Health Sciences Center, West Virginia University, Morgantown, West Virginia, United States of America; 8 Institute of Pathology, University Medical Center Freiburg, Freiburg, Germany; 9 Translational Hepatology and Stem Cell Biology, REBIRTH cluster of excellence, Hannover Medical School, Hannover, Germany; 10 Institute for Cellular and Molecular Pathology, Hannover Medical School, Hannover, Germany; The Jackson Laboratory, UNITED STATES

## Abstract

*In vitro* disease modeling based on induced pluripotent stem cells (iPSCs) provides a powerful system to study cellular pathophysiology, especially in combination with targeted genome editing and protocols to differentiate iPSCs into affected cell types. In this study, we established zinc-finger nuclease-mediated genome editing in primary fibroblasts and iPSCs generated from a mouse model for radiosensitive severe combined immunodeficiency (RS-SCID), a rare disorder characterized by cellular sensitivity to radiation and the absence of lymphocytes due to impaired DNA-dependent protein kinase (DNA-PK) activity. Our results demonstrate that gene editing in RS-SCID fibroblasts rescued DNA-PK dependent signaling to overcome radiosensitivity. Furthermore, *in vitro* T-cell differentiation from iPSCs was employed to model the stage-specific T-cell maturation block induced by the disease causing mutation. Genetic correction of the RS-SCID iPSCs restored T-lymphocyte maturation, polyclonal V(D)J recombination of the T-cell receptor followed by successful beta-selection. In conclusion, we provide proof that iPSC-based *in vitro* T-cell differentiation is a valuable paradigm for SCID disease modeling, which can be utilized to investigate disorders of T-cell development and to validate gene therapy strategies for T-cell deficiencies. Moreover, this study emphasizes the significance of designer nucleases as a tool for generating isogenic disease models and their future role in producing autologous, genetically corrected transplants for various clinical applications.

## Author Summary

Due to the limited availability and lifespan of some primary cells, *in vitro* disease modeling with induced pluripotent stem cells (iPSCs) offers a valuable complementation to *in vivo* studies. The goal of our study was to establish an *in vitro* disease model for severe combined immunodeficiency (SCID), a group of inherited disorders of the immune system characterized by the lack of T-lymphocytes. To this end, we generated iPSCs from fibroblasts of a radiosensitive SCID (RS-SCID) mouse model and established a protocol to recapitulate T-lymphopoiesis from iPSCs *in vitro*. We used designer nucleases to edit the underlying mutation in *prkdc*, the gene encoding DNA-PKcs, and demonstrated that genetic correction of the disease locus rescued DNA-PK dependent signaling, restored normal radiosensitivity, and enabled T-cell maturation and polyclonal T-cell receptor recombination. We hence provide proof that the combination of two promising technology platforms, iPSCs and designer nucleases, with a protocol to generate T-cells *in vitro*, represents a powerful paradigm for SCID disease modeling and the evaluation of therapeutic gene editing strategies. Furthermore, our system provides a basis for further development of iPSC-derived cell products with the potential for various clinical applications, including infusions of *in vitro* derived autologous T-cells to stabilize patients after hematopoietic stem cell transplantation.

## Introduction

Studying the molecular pathology of human disease is often difficult due to the limited availability of particular primary cells, their limited lifespan, or because complex developmental differentiation procedures cannot be easily followed *in vivo*. *In vitro* disease modeling with induced pluripotent stem cells (iPSCs) provides a practical alternative, and the study of several disorders has benefitted enormously from the convergence of three key technologies: modern genomics that links genetic variants to disease phenotypes, the ability to generate patient-specific iPSCs that can be differentiated into cell types affected by disease, and powerful tools for editing complex genomes [1,2].

T lymphocytes play an important role in adaptive immunity against invading pathogens or in fighting tumor cells. A natural microenvironment for T-cell lymphopoiesis is provided by the thymus. Inherited defects in T-cell function or in T-cell development can lead to severe combined immunodeficiency (SCID), a group of life threatening disorders of the immune system [3]. Radiosensitive SCID (RS-SCID; OMIM #602450) is characterized on the molecular level by dysfunctional non-homologous end-joining (NHEJ), the most important pathway to repair DNA double strand breaks (DSBs). In human patients, defective DNA repair can lead to a cellular hypersensitivity to ionizing radiation. Moreover NHEJ is essential for physiological B- and T-lymphocyte development as it plays an important role in the B-cell receptor (BCR) and T-cell receptor (TCR) recombination process [4]. The diversity of BCRs and TCRs results from the multitude of variable (V), divers (D) and joining (J) gene segments that are almost randomly reassembled in a process called V(D)J recombination. During V(D)J recombination, specific enzymes cleave at specific recombination signal sequences flanking these gene segments and NHEJ factors play a crucial role in reassembly and final ligation of these gene segments [5,6]. The NHEJ process involves a number of different enzymes, including DNA-dependent protein kinase (DNA-PK). DNA-PK is a polyprotein complex, formed by the Ku70/Ku80 heterodimer and the DNA-PK catalytic subunit (DNA-PKcs) [7], that binds to DNA end structures and serves as a docking site for additional NHEJ factors that mediate DNA repair [8]. Hypomorphic mutations in *PRKDC*, the locus encoding DNA-PKcs, have recently been described for radiosensitive T and B deficient SCID patients [9]. Hence, DNA-PK dependent signaling is a paradigmatic example of how a single molecule can be simultaneously involved in both, DNA repair and T- and B-cell development, and of how such a process can be disturbed by a single point mutation. These particularities make *PRKDC* an optimal target for novel site-specific gene therapy approaches, such as designer nuclease mediated genome editing.

For disease modeling, iPSCs can be generated from affected somatic cells by expression of four transcription factors Oct4, Sox2, Klf4 and c-Myc [10,11]. Similar to pluripotent embryonic stem cells, iPSCs have the capacity for unlimited self-renewal, are permissive for transfection with foreign DNA, and importantly, can be expanded in a clonal fashion for characterization. Thus far, iPSCs have been derived from several patients suffering from different hematopoietic and immunological disorders and have been used for disease modeling and gene targeting approaches [12]. Several protocols for *in vitro* [13–21] and *in vivo* [22,23] differentiation of iPSCs to hematopoietic cells have been reported. The availability of Notch ligand based cell culture systems, such as the murine stromal cell line OP9-DL1, allows for further differentiation of hematopoietic stem cells into T-cells *in vitro* [24,25]

Targeted genome modification in iPSCs is an essential tool in disease modeling [12], and gene editing with designer nucleases has developed into a powerful instrument, which has been successfully applied to generate various genetically modified model organisms or human cells to study gene function or the pathophysiology of disease causing mutations. Designer nucleases, like meganucleases [26], zinc-finger nucleases (ZFNs) [27], transcription activator-like effector nucleases (TALEN) [28], or the clustered regularly interspaced short palindromic repeats (CRISPR)/Cas9 system [29], induce site-specific DNA double strand breaks (DSBs) at chosen sites. These DSBs activate one of two major DNA repair mechanisms, NHEJ or homology directed repair (HDR), which can be employed to disrupt genes or to target the integration of exogenous donor DNA sequences to a specific site in the genome, respectively [30].

The goal of this study was to establish an *in vitro* disease model for T-cell deficiencies and to employ this model to evaluate a designer nuclease-based genome editing strategy. To this end, we generated iPSCs from adult ear fibroblasts of NOD.SCID mice, a model for RS-SCID [31], and established a protocol to recapitulate T-lymphopoiesis from iPSCs *in vitro*. We used ZFNs to edit DNA-PK deficient fibroblasts and iPSCs and demonstrated that designer nuclease mediated gene correction led to rescue of DNA-PK dependent signaling, normal radiosensitivity, restoration of T-cell maturation, and polyclonal TCR recombination. We hence provide proof that the combination of two promising technology platforms, iPSCs and designer nucleases, with a protocol to generate T-cells *in vitro* represents a powerful paradigm for SCID disease modeling and the evaluation of therapeutic gene editing strategies.

## Results

### Restoring DNA-PK activity in SCID fibroblasts by targeted genome editing

**Fig 1:**
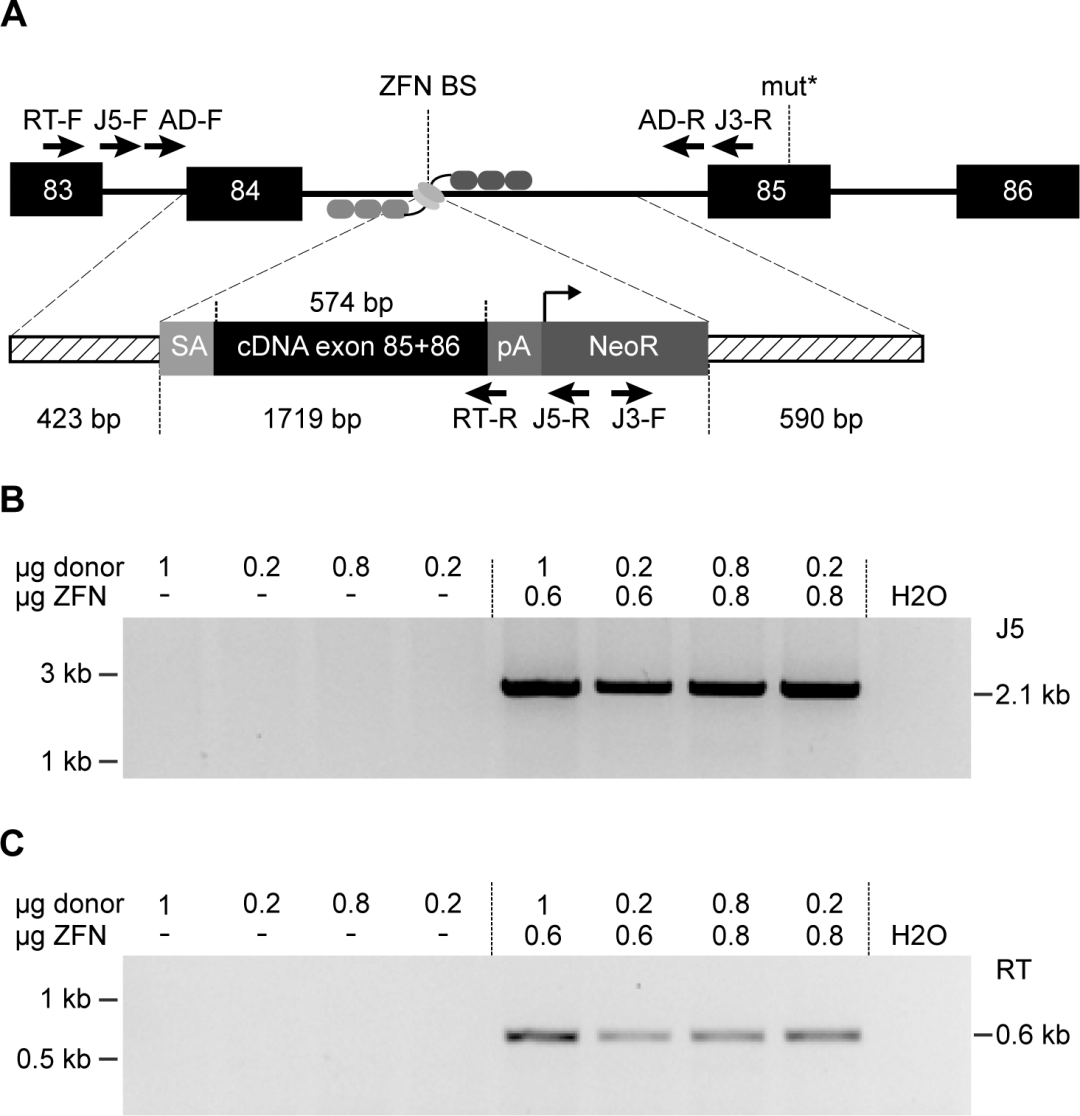
Targeted genome editing in RS-SCID fibroblasts. (**A**) Schematic of genome editing strategy. Homology-directed repair (HDR) between the *prkdc* locus and the donor DNA is promoted by ZFN cleavage in intron 84 (BS, binding site). The HDR donor consists of flanking homology arms (dashed lines), splice acceptor (SA), cDNA encoding *prkdc* exons 85 and 86, polyadenylation signal (pA), neomycin resistance cassette (*NeoR*). The SCID underlying mutation in exon 85 (mut*), and primer binding sites for PCR analysis (5’-junction J5-F/J5-R; 3’-junction J3-F/J3-R; allelic discrimination AD-F/AD-R; mRNA expression RT-F/RT-R) are indicated. (**B**) Genome editing. After transfection of SCID fibroblasts with various ratios of donor DNA to ZFN expression plasmids, successful gene targeting in polyclonal samples was detected by an inside-out PCR amplification of the genome–donor 5´-junction (J5-F/J5-R). (**C**) Expression of corrected *prkdc* mRNA. After transfection of SCID fibroblasts, successful splicing from exon 83 to cDNA was detected with an inside-out RT-PCR strategy using primers RT-F/RT-R.

In the murine disease model RS-SCID is caused by a T-to-A transversion mutation in exon 85 of the *prkdc* locus. The introduced premature stop codon (Y4046*) leads to an 83 aa long C-terminal truncation of the encoded DNA-PKcs protein, leading to decreased protein stability and low kinase activity [31]. ZFNs targeted to intron 84 of *prkdc* were generated using the OPEN platform [32] and their activity verified by *in vitro* cleavage assays and plasmid-based recombination assays (S1 Fig). To restore function of DNA-PK, we generated a donor DNA encompassing the wild-type cDNA sequence of *prkdc* exons 85 and 86, preceded by a splice acceptor site and followed by a poly(A) signal (Fig 1A). Targeting an intron allowed us to co-introduce a neomycin selection marker cassette to enrich for cells that underwent correct gene targeting.

To validate our targeting strategy, fibroblasts from a 12-week old male NOD.SCID mouse, in which the SCID mutation in *prkdc* was confirmed by sequencing, were isolated. Upon culturing *in vitro* these cells transformed spontaneously, probably due to their intrinsic DNA repair deficiency. The fibroblasts were transfected with various ratios of donor DNA to ZFN expression plasmids before G418 selection was applied. An inside-out PCR strategy was used to verify correct gene targeting in polyclonal cell populations (Fig 1B). All samples transfected with ZFN expression plasmids and donor revealed successful gene targeting. Splicing of exon 84 to the integrated cDNA was verified by inside-out reverse transcription (RT)-PCR (Fig 1C). To determine the efficiency of the gene targeting approach, cell clones were generated by single cell dilution. Six out of 20 analyzed clones showed correct targeting.

**Fig 2:**
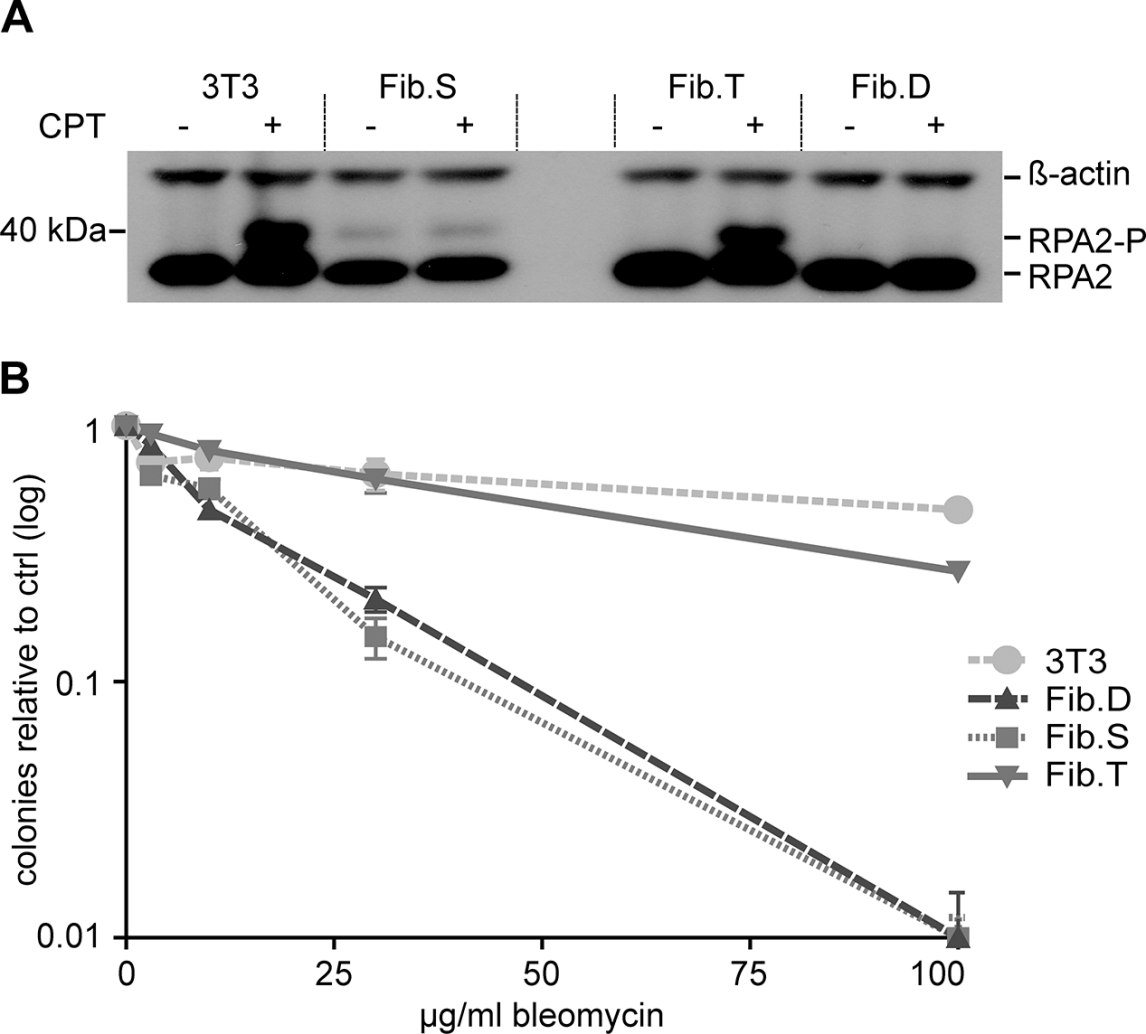
Functional correction of RS-SCID fibroblasts. (**A**) DNA-PK dependent phosphorylation of RPA2. Treatment of fibroblasts with camptothecin (CPT) induces DNA-PK dependent phosphorylation of RPA2, which was detected by Western blot analysis using an RPA2 specific antibody. Detection of ß-actin served as a loading control. Positions of RPA2 and its phosphorylated form, RPA2-P, are indicated on the right. (**B**) Rescue of radiosensitivity. Fibroblasts were cultured with increasing amounts of the radiomimetic drug bleomycin. Cellular sensitivity to the drug was quantified by counting number of surviving colonies relative to untreated samples. Data are represented as mean ± SD (N = 3). 3T3, NIH-3T3 fibroblasts; Fib.S, SCID fibroblasts; Fib.T, gene targeted SCID fibroblasts; Fib.D, fibroblasts treated with randomly integrated donor.

To confirm that re-routed splicing of exon 84 to the artificial exon 85/86 restored DNA-PK activity, cells were treated with camptothecin (CPT), a compound known to induce DSBs during DNA replication by blocking topoisomerase I. Under these experimental conditions, RPA2 is exclusively phosphorylated by DNA-PK at the stalled replication forks [33]. Upon CPT treatment of SCID fibroblasts (Fib.S), a gene edited fibroblast clone (Fib.T) and a donor-containing clone (Fib.D), phosphorylation of RPA2 was detected in Fib.T cells, but not in Fib.S and Fib.D cells (Fig 2A). The fibroblast cell line NIH-3T3 served as a positive control.

Fibroblasts of RS-SCID mice are sensitive to gamma-irradiation or the radiomimetic drug bleomycin [34]. To verify that successful gene targeting could abrogate radiosensitivity, colony survival assays with bleomycin were conducted. We found that the corrected cell line Fib.T displayed similar resistance to the drug as NIH-3T3 cells, while both Fib.D and Fib.S cells were highly sensitive to bleomycin (Fig 2B). In conclusion, successful ZFN-mediated genome editing restored activity of DNA-PK, which was able to phosphorylate downstream target proteins and to rescue the radiosensitive phenotype of RS-SCID cells.

### Genetic correction of SCID iPSCs

**Fig 3:**
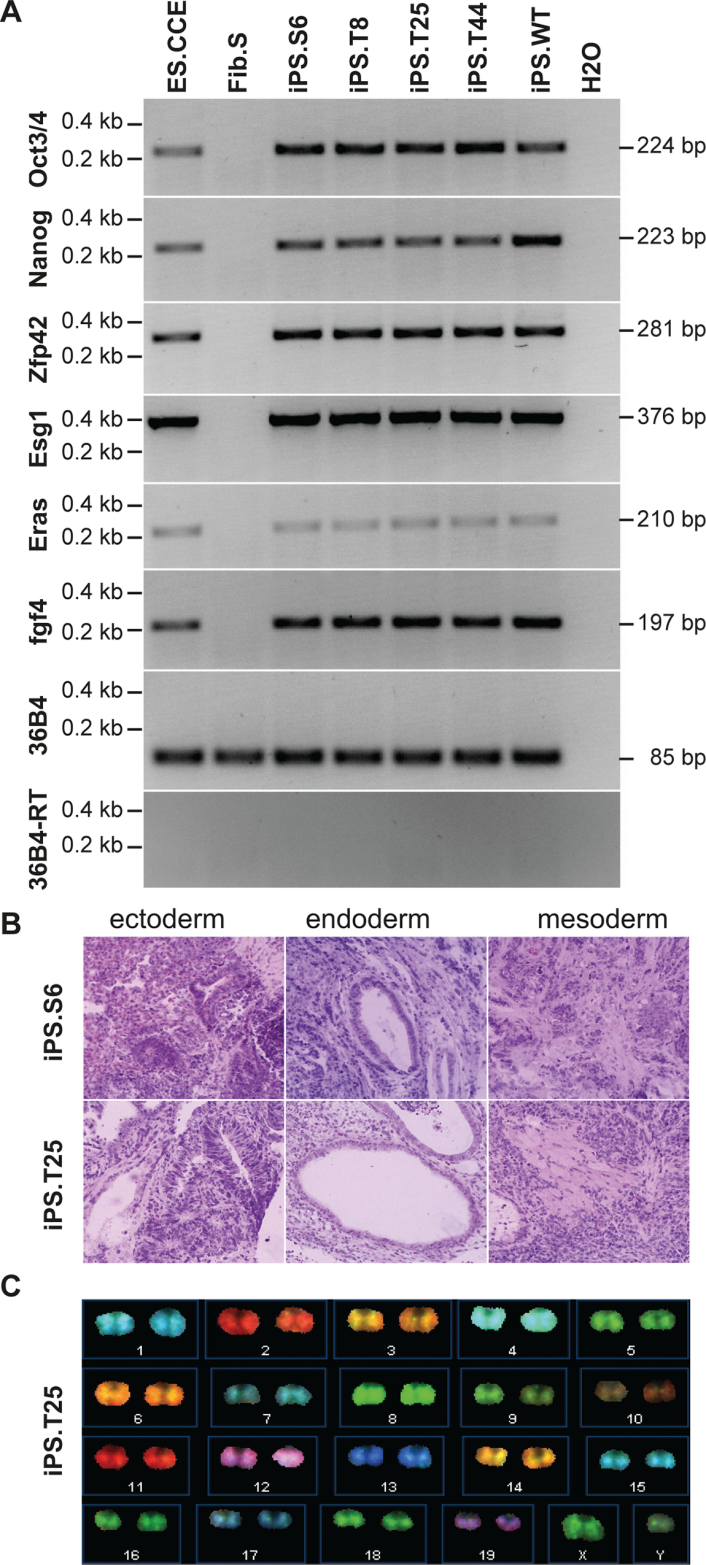
Evaluation of pluripotency of generated iPSCs. (**A**) Pluripotent stem cell marker gene expression. Oct3/4, Nanog, Zfp42, Esg1, Eras, and fgf4 mRNA expression was determined by qualitative RT-PCR (see S2 Table). Housekeeping gene 36B4 (+/- reverse transcriptase) were included as controls. ES.CCE, murine embryonic stem cell line; Fib.S, SCID fibroblasts; iPS.S6, SCID iPSC clone; iPS.T8, iPS.T25, iPS.T44, gene targeted SCID iPSC clones; iPS.WT, wild-type iPSC clone. (**B**) *In vivo* differentiation analysis. Teratoma formation was induced by subcutaneous injection of iPSCs into mice. **Hematoxylin/eosin**-stained sections of teratoma-derived from clones iPS.S6 and iPS.T25 are shown. (**C**) Karyotype analysis. Spectral karyotyping (SKY) was performed to detect microscopic genomic abnormalities, translocations and aneuploidies in untreated or genetically corrected SCID iPSC clones. SKY analysis of clone iPSC.T25 is shown (see also S2 Fig).

While fibroblasts served as an important model to evaluate DNA-PK dependent signaling, the full therapeutic potential of genome editing at the *prkdc* locus can only be assessed in lymphoid cells. iPSCs have the capacity for unlimited self-renewal, allowing long-term *in vitro* culture and generation of single-cell derived subclones. As iPSCs can be differentiated into hematopoietic cells, including T lymphocytes, they are an ideal platform for disease modeling and the evaluation of gene therapeutic approaches. We generated iPSCs from fibroblasts isolated from a 6-week-old NOD.SCID mouse by transduction with a polycistronic lentiviral vector expressing the reprogramming factors Oct4, Klf4, Sox2 and c-Myc [35]. Since the DNA repair-deficient phenotype interferes with efficient reprogramming [36], we conducted the experiment under hypoxic conditions and added ascorbic acid to reduce damage by reactive oxygen species (ROS) [37]. In addition, small molecule inhibitors for MAP kinase (MEK), glycogen synthase kinase 3 (GSK3) and TGF-beta were used, which have been reported to permit derivation of iPSCs of NOD-derived mouse strains and enhance the reprogramming progress [38,39]. All analyzed iPSC clones expressed pluripotent stem cell markers (S2 Fig), and RT-PCR demonstrated expression of the embryonic stem cell-specific genes in a NOD.SCID iPSC clone (iPS.S6; Fig 3A). In addition, cells from ectodermal (neural rosette-like structures), endodermal (gut-like structures) and mesodermal (smooth muscle patches) origin were detected in teratoma derived from clone iPS.S6 (Fig 3B). Genome integrity was assessed before and after ZFN mediated genome engineering by spectral karyotyping (Figs 3C and S2). The NOD.SCID iPSC clone iPS.S6 displayed no gross genetic abnormalities and was used for subsequent gene targeting experiments. In summary, we showed that DNA-repair deficient NOD.SCID fibroblasts could be reprogrammed into iPSCs that display pluripotent behavior and characteristics similar to murine embryonic stem cells.

**Fig 4:**
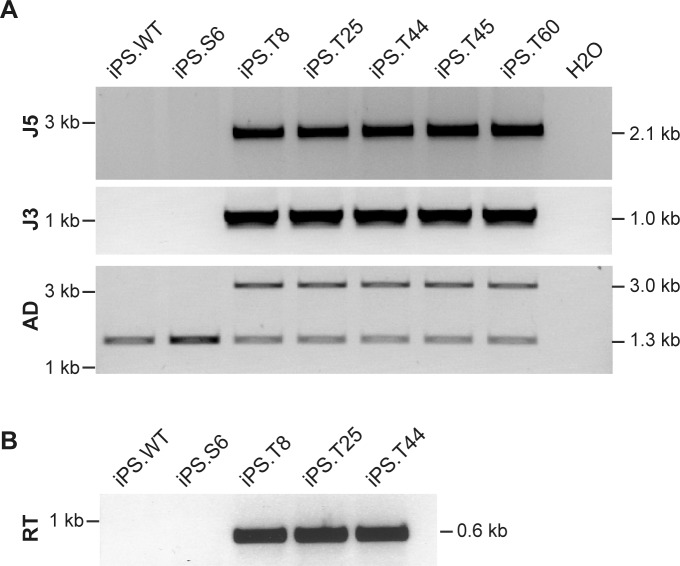
Targeted genome editing in SCID-derived iPSCs. (**A**) Verification of gene targeting. Inside-out PCR strategies (see Fig 1A) were used to verify correct 5´ (J5) and 3´-junctions (J3) of the integrated donor. Allelic discrimination (AD) PCR was used to assess mono- vs. bi-allelic integration. Targeted allele runs at 2.99 kb. Sizes of all expected PCR amplicons are indicated on the right. iPS.WT, wild-type iPSC; iPS.S6, SCID iPSC clone; iPS.T8, iPS.T25, iPS.T44, iPS.T45 and iPS.T60, targeted SCID iPSC clones. (**B**) Expression of corrected *prkdc* mRNA. Successful splicing from exon 83 to cDNA encompassing exons 84/85 was detected by an inside-out RT-PCR strategy using primers RT-F/RT-R.

For targeted genome editing, cells of iPSC clone iPS.S6 were nucleofected with donor and ZFN expression plasmids. Following selection and clonal expansion, inside-out PCR amplification was applied on genomic DNA to detect correct targeting. Of note, 41 out of 46 analyzed clones (89%) showed correct integration of the artificial exon 85/86. Extended PCR analysis of five targeted iPSC clones verified correct 5´- and 3´-junctions between genomic and donor DNA, respectively. An allelic discrimination PCR confirmed mono-allelic targeting in all cases (Fig 4A). Furthermore, expression of the DNA-PKcs encoding mRNA and re-routed splicing to artificial exon 85/86 was validated by inside-out RT-PCR (Fig 4B). All targeted iPSC clones were positive for expression of pluripotency markers (Fig 3A), formed all three germ layers in teratoma assays (Figs 3B and S2), had an intact karyotype (Figs 3C and S2), and did not show any signs of NHEJ-mediated mutagenesis at the top 15 predicted off-target sites in the mouse genome (S1 Table, S1 Text).

The polycistronic lentiviral vector used for generation of iPSCs contained Flp recognition target (FRT) sites in the U3 region of the long-terminal repeats, which allowed us to excise the reprogramming cassette using retroviral-mediated transfer of Flp recombinase [40]. Southern blot analysis confirmed successful removal of the lentiviral vector genome (S3 Fig) and targeted integration of the artificial exon 85/86 into intron 84 of the *prkdc* locus (S3 Fig).

### Differentiation of iPSCs to TCR-positive T lymphocytes

**Fig 5:**
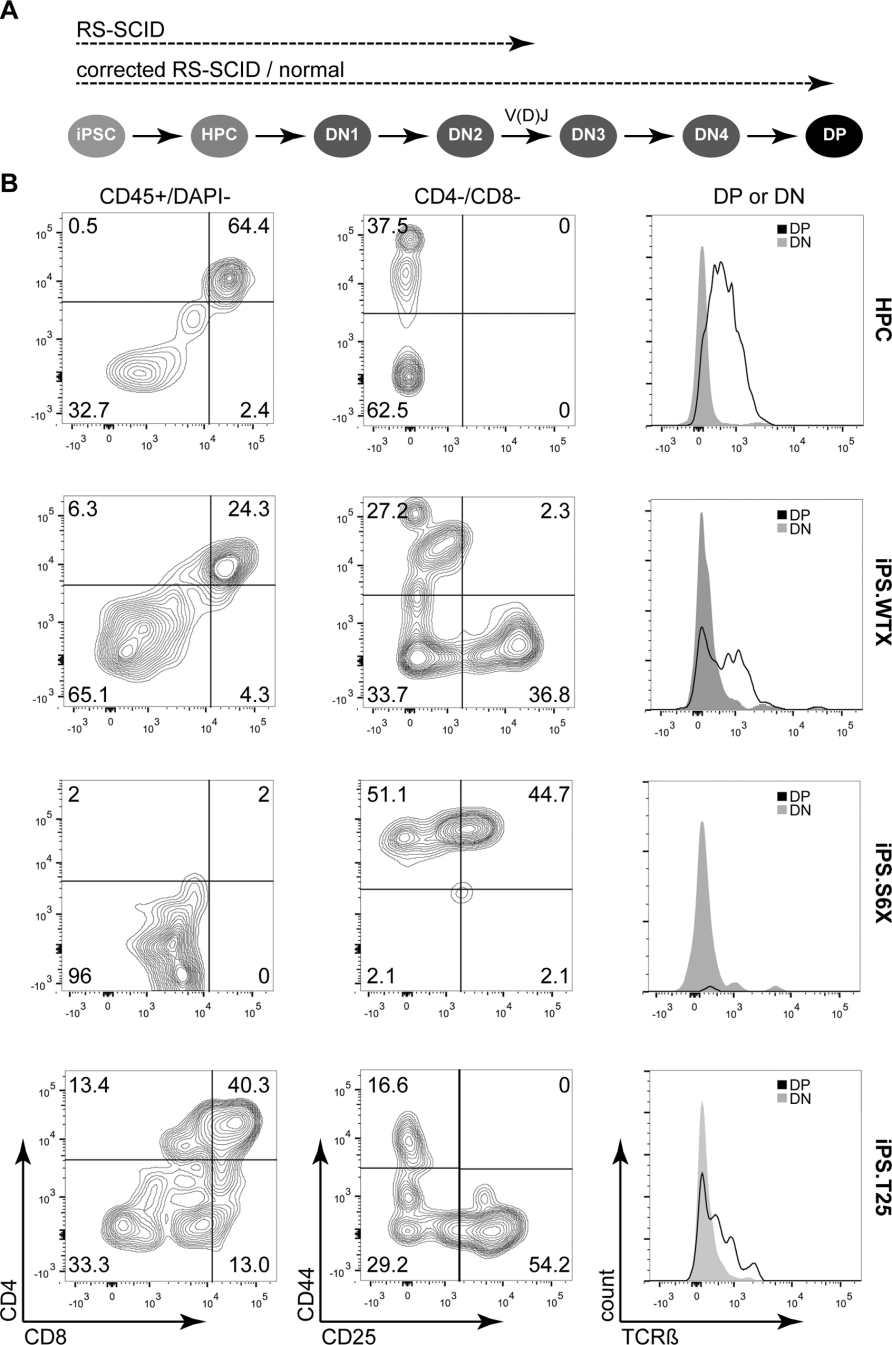
*In vitro* differentiation of iPSCs to proT-cells and T-cells. (A) Schematic of *in vitro* T-cell differentiation from iPSCs. Differentiation of iPSCs starts with formation of embryoid bodies that are dissociated to give rise to hematopoietic stem and progenitor cells (HPC). DL-1 mediated Notch signaling coaxes HPC development towards early proT-cells (DN2), which undergo DNA-PK dependent V(D)J recombination. After passing through DN3 and DN4 stages, preT-cells mature into double-positive (DP) T-cells that express the beta chain of the T-cell receptor (TCRß). Dashed lines indicate to what stage iPSC clones are expected to differentiate. (B) Assessment of T-cell differentiation. *In vitro* T-cell differentiation was analyzed by flow cytometry after two weeks of co-cultivation on OP9-DL1. Gating (indicated on top of each column) was applied in the following order: FSC/SSC and CD45^+^/DAPI^—^to assess CD4/CD8 expression; CD8^–^/CD4^—^to gate for DN1-DN4 stage cells; CD8^–^/CD4^–^ (DN) or CD8^+^/CD4^+^ (DP) to assess TCRß expression. Numbers indicate percentage of cells in each quadrant. HPC, lineage-negative bone marrow cells; iPS.WTX, wild-type iPSC; iPS.S6X, SCID iPSC clone; iPS.T25, gene targeted SCID iPSC clone.

Since DNA-PK is essential for V(D)J recombination, the RS-SCID immunophenotype is characterized by a lack of T and B-lymphocytes [41]. The stromal cell line OP9-DL1 leads to activation of the DL1-mediated Notch signaling in co-cultured cells, which in turn is a prerequisite to induce the T-lymphoid program in multipotent hematopoietic progenitors [24]. Initial experiments showed that OP9-DL1 co-cultivation of C57BL/6-derived lineage negative bone marrow cells enabled the differentiation of these multipotent stem cells through all (CD4^-^/CD8^-^) double-negative (DN) thymocyte stages, as determined by CD25 and CD44 surface expression. Further culturing of these T-cell precursors on OP9-DL1 led to the generation of CD4^+^/CD8^+^ double-positive (DP) T lymphocytes, which expressed the beta chain of the T-cell receptor (TCRß), indicating that these cells have successfully undergone V(D)J recombination and beta-selection *in vitro* (Fig 5).

Since V(D)J recombination is initiated at the DN2 (CD44^+^/CD25^+^) stage and beta-selection occurs at the DN3 (CD44^-^/CD25^+^) stage, we hypothesized that corrected RS-SCID iPSC-derived hematopoietic progenitor cells (HPCs) should be able to differentiate to CD4^+^/CD8^+^ double-positive T lymphocytes, while T-cells derived from uncorrected SCID-derived iPSCs would stop at the DN2 thymocyte stage due to their defect in V(D)J recombination (Fig 5A). To this end, we established an embryoid body (EB)-based differentiation protocol for the generation of HPCs from iPSCs. Differentiated and dissociated EBs from all iPSC clones contained cells carrying the early hematopoietic surface markers CD41 and cKit (S4 Fig). Co-cultivation of these cells on OP9-DL1 stroma cells induced differentiation towards T-lymphocytes. After two weeks, thymocyte maturation of iPSC-derived HPCs was measured by flow cytometry, revealing the presence of CD44^+^/CD25^-^ (DN1), CD44^+^/CD25^+^ (DN2), CD44^-^/CD25^+^ (DN3), CD44^-^/CD25^-^ (DN4), and CD4^+^/CD8^+^ (DP) cells from wild-type iPSCs (iPS.WTX; Fig 5). As hypothesized, T-cell differentiation of NOD.SCID iPSCs (iPS.S6X) was blocked in early DN1 and DN2 thymocyte stages and these T-cell precursors showed neither expression of CD4/CD8 nor TCRß. In contrast, differentiation from genetically corrected iPSC clones (iPS.T25X) reached DN3 and DN4 stages as well as the CD4^+^/CD8^+^ DP T-cell stage, with a fraction of cells expressing TCRß (Fig 5B). Although the same experimental conditions were applied, the absolute numbers of generated T-cells varied in between different experiments.

**Fig 6:**
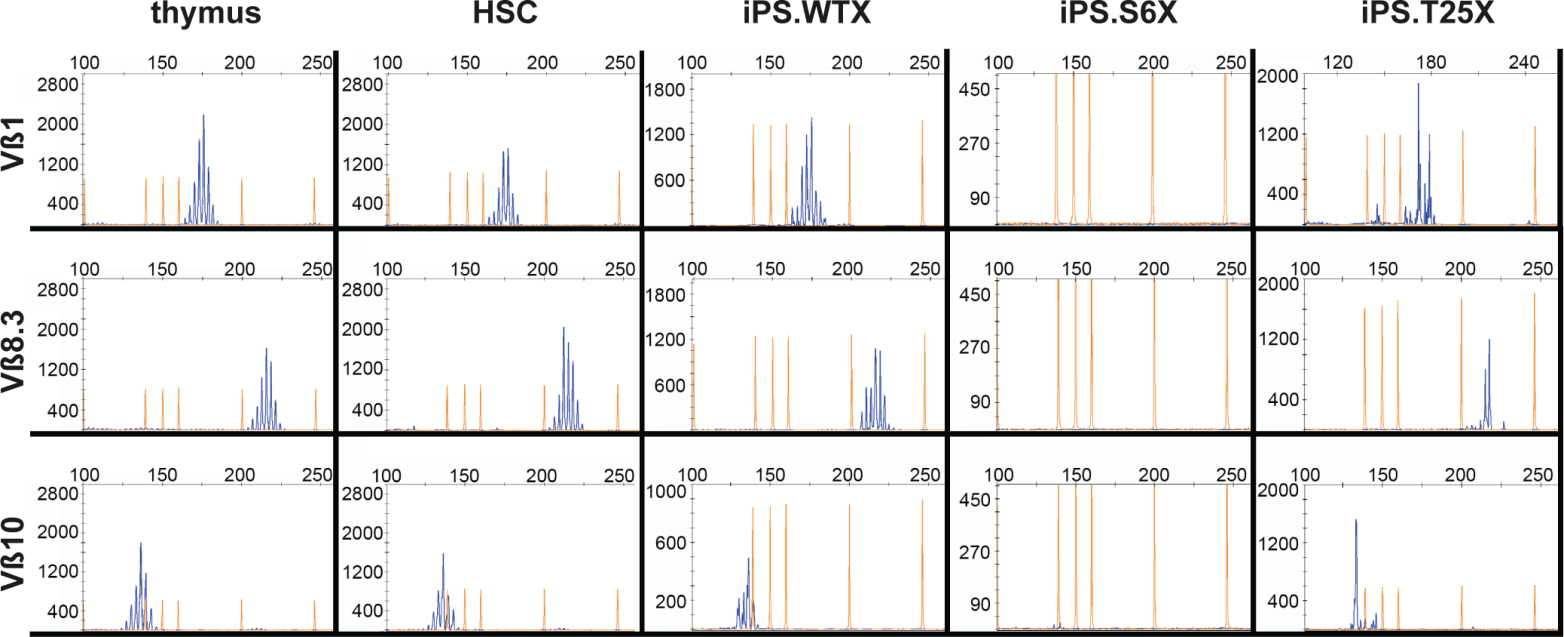
Polyclonal T-cell receptor recombination. *In vitro* generated T-cells were analyzed by spectratyping, i.e. quantitative RT-PCR expression analysis of the variable beta chains. Exemplarily shown are results for Vß1, Vß8.3 and Vß10 (see also S5 Fig). X-axis depicts detected PCR fragment size in bp, Y-axis depicts counts of obtained PCR fragments. Thymus, T-cells isolated from thymus as a positive control; HSC, *in vitro* generated T-cells from lineage-negative bone marrow cells; iPS.WTX, wild-type iPSCs; iPS.S6X, SCID iPSC clone; iPS.T25X, gene targeted SCID iPSC clone.

To confirm T-cell receptor recombination on the genome level, V(D)J recombination was verified by spectratyping. Control T-cells isolated from the thymus and *in vitro* generated T-cells from bone marrow lineage negative cells showed a polyclonal T-cell repertoire at Vß chains 1, 6, 8.1, 8.3, 10, 12, 14 and 20 (Figs 6 and S5). While V(D)J recombination was undetectable in T-cell precursors derived from SCID iPSCs, T lymphocytes derived from WT or gene targeted iPSCs underwent V(D)J recombination and revealed a polyclonal T- cell repertoire.

In summary, we developed a protocol, which allowed us to model T-cell differentiation *in vitro*. We showed that iPSCs can be differentiated into hematopoietic progenitors and further to various stages of thymocyte development. While wild-type and corrected NOD.SCID iPSCs could be maturated into CD45^+^ CD4^+^/CD8^+^ DP T-cells that express TCRß, differentiation of DNA-PK-deficient cells stopped at the DN2 thymocyte stage. These results provide a proof of concept that iPSC-based *in vitro* disease modeling is able to reflect *in vivo* thymocyte maturation and that such modeling can be used for both to investigate T-cell maturation defects and to validate gene therapy strategies.

## Discussion

SCID is a group of monogenetic disorders of the immune system characterized by the absence of T-cells, sometimes in combination with a lack of functional B-lymphocytes and/or natural killer cells. RS-SCID is a special form of SCID disorders and serves as a paradigm for radiosensitivity and immunodeficiency. On top of the absence of T- and B-lymphocytes, the pathophysiology of RS-SCID is characterized by a strong sensitivity of all somatic cells to radiation and DNA damaging agents due to a defective DNA repair pathway. The underlying mutations are found in genes coding for NHEJ factors, including LIG4 [42], Artemis [43], XLF [44] and DNA-PKcs [9].

Disease modeling based on patient-derived iPSCs is particularly valuable when studying rare disorders, like RS-SCID, for which patient cells are not easily accessible, have a limited lifespan, or do not develop due to a differentiation block. Designer nuclease-based gene editing in iPSCs makes this instrument even more attractive because it enables scientists to correlate genotype to phenotype in an isogenic background, either by creating disease models through the insertion of disease specific mutations in normal cells [45] or by correcting the underlying genetic mutation back to wild-type in patient-derived iPSCs [16,46]. Particularly in combination with genetic engineering, iPSCs are preferred over fibroblasts because of their unlimited proliferative potential and their ability of clonal expansion.

Hematopoietic differentiation protocols offer the possibility to investigate maturation of various blood lineages *in vitro*, e. g. to study the impact of genomic mutations on protein function in mature blood cells or where specific mutations lead to a block in lymphopoiesis, myelopoiesis, or erythropoiesis [17,18,46]. While designer nuclease-based gene editing in iPSCs has been established in several labs, differentiation of genetically modified iPSCs to mature immune cells has remained challenging. Differentiation of iPSCs derived from a patient suffering from X-linked chronic granulomatous disease (X-CGD) to granulocytes was the first example to show functional correction of a genetic defect by targeted integration of a gp91^phox^ expression cassette into the putative safe harbor site *AAVS1* [15]. Myeloid differentiation from patient-derived iPSCs for disease modeling and/or drug development has also been established e.g. for severe congenital neutropenia [47] and pulmonary alveolar proteinosis [20].

Differentiation of iPSCs to lymphocytes, on the other hand, has been reported only from a few labs [18,19]. In the present study, we describe an improved *in vitro* differentiation procedure for iPSCs to T-cells that is based on previously published protocols [18,48,49], and, to our knowledge, use this protocol for the first time to model the functional defects of an immunodeficiency *in vitro* and to investigate the effect of genetic engineering of disease iPSCs on T-cell maturation. Because the generated hematopoietic progenitor cells supported the maturation through all early stages of thymocyte differentiation, including V(D)J recombination and beta-selection, we were able to reproduce the stage-specific block induced by the point mutation in the *prkdc* locus *in vitro*. This setup can also be used to screen for genotype-phenotype correlations or to characterize the consequence of newly identified genetic mutations on T-lymphopoiesis and/or T-lymphocyte function in more detail. For instance, as compared to *in vivo* models, individual effects of the microenvironment, cytokines and/or small molecules affecting T-cell maturation and expansion, like IL-7 or IL-2, can be analyzed by simple addition to the culture medium. Moreover, existing stroma-free models can be further developed [50] to identify factors downstream of Delta-like Notch ligands that promote T-cell development. Finally, the efficiency of T-cell related gene therapy approaches can be assessed *in vitro*, without the need of hematopoietic stem cells of the patients.

In our study we applied ZFNs for genetic modification of RS-SCID iPSCs. The generation of highly specific ZFNs can be rather challenging and several studies have described off-target cleavage activity of ZFNs [51,52]. While the specificity of ZFNs can be improved, e.g. by optimizing the DNA binding properties of the zinc-finger arrays [32], selecting appropriate linker domains [53,54] and employing obligate heterodimeric *Fok*I nuclease domains [55,56], alternative designer nucleases, such as TALENs [28] and CRISPR/Cas9 based nucleases [29], are easier to engineer.

Our system provides a basis for further development of iPSC-derived cell products with the potential for various clinical applications. However, although we have tried to transplant iPSC-derived hematopoietic stem/precursor cells into NOD.SCID mice, we did not observe any engraftment of these cells. This is in line with published data showing that transplantation worked only with iPSC-derived hematopoietic stem/precursor cells that were produced *in vivo* [22,23]. Further studies will be needed to establish optimal culture conditions to generate transplantable stem cells *in vitro*. Hence, combining *in vitro* protocols with physiologic *in vivo* differentiation seems more promising. For example, transplantation of iPSC-derived early thymocyte progenitor populations could allow for thymic reconstitution and maturation to create polyclonal T-cell effector populations [50]. Infusions of *in vitro* derived autologous T-cells could be used to stabilize patients suffering from primary immunodeficiencies, like SCID or hemophagocytic lymphohistiocytosis, or after conventional hematopoietic stem cell transplantation to close the gap until graft-derived lymphocytes arise. Moreover, given the clinical success of autologous T-cells expressing tumor specific chimeric antigen receptors (CARs) [57], iPSC-derived autologous CAR-T-cells represent an interesting alternative to current protocols, as recently shown [19]. Finally, autologous, CCR5 knockout iPSC lines could present a source to provide HIV patients with HIV-resistant T-cells to reconstitute the adaptive immune system [58]. However, before iPSC-based cell therapies can enter clinical practice, safety concerns, especially with regard to the generation of iPSC-derived teratoma, have to be addressed and full functionality of iPSC-derived cells proven.

In conclusion, our study describes an iPSC-based disease model for RS-SCID. Our *in vitro* protocol allowed us to differentiate iPSCs to T-cells and to analyze the influence of NHEJ deficiency on V(D)J recombination. Moreover, it emphasizes the significance of designer nucleases as a tool in generating isogenic disease models and their future role in producing iPSC-based, patient-specific, genetically corrected autologous transplants for various applications in the clinic.

## Materials and Methods

### Cells and cell culture

NIH.3T3 and HEK293T cells were cultured in DMEM (Biochrom) supplemented with 10% FCS (PAA), penicillin/streptomycin (P/S; PAA), L-glutamine (Biochrom) and sodium pyruvate (PAA). OP9 and OP9-DL1 cells (obtained from Juan Carlos Zúñiga-Pflücker) were expanded in OP9 medium [alpha-MEM (Gibco), 20% OP9-tested FCS (PAA), P/S and L-glutamine]. Primary mouse ear fibroblasts were cultured in MEF medium [DMEM low glucose (PAA) with 15% FCS, L-glutamine, nonessential amino acids (NEAA; Gibco), P/S, 100 μM of ß-mercaptoethanol (Sigma-Aldrich), sodium pyruvate and 50 μg/μl phospho-ascorbic acid (P-VitC, Sigma-Aldrich)]. ES.CCE cells were cultivated in ES medium [Knockout-DMEM (Gibco) with 15% ES-tested FCS (PAA), P/S, L-glutamine, NEAA, 150 mM monothioglycerol (MTG, Sigma-Aldrich) and ESGRO mouse LiF (Millipore)]. iPSCs were cultivated in iPS medium [Knockout-DMEM supplemented with 15% ES-tested FCS, NEAA, P/S, L-Glutamine, 100 μM of ß-mercaptoethanol and ESGRO mouse LiF, 50 μg/μl of P-VitC, 4 μM of SB431542, 1 μM of PD0325901 and 3 μM of CHIR99021 (all Axon Medchem, together termed 3i) and passaged with Accutase (Gibco). ES.CCE cells and iPSCs were cultivated either on irradiated C3H or CF-1 MEF feeders on gelatin-coated plates or feeder-free in vented flasks (Sarstedt). Lineage negative cells (HSC) were isolated by flushing the tibiae and femurs of C57BL/6N mice (Charles River) and purified by magnetic cell sorting (MACS) with the Lineage Cell Depletion Kit (MACS Miltenyi) according to the manufacturer’s protocol. Cells were stained with Trypan Blue (Sigma-Aldrich) and counted at 100x microscope magnification prior to *in vitro* T-cell differentiation. Cell clones were generated either by limiting dilution (fibroblasts) or colony picking (iPSCs). All but HEK293T cells were cultivated under hypoxic conditions (7% CO_2_ / 5% O_2_).

### Plasmids

*Prkdc*-specific zinc-finger arrays (S1 Fig) were generated with the OPEN protocol [32]. To generate ZFNs, the zinc-finger arrays were codon-optimized (GeneArt) and cloned into pRK5 vectors, with and without NLS [59], containing the cleavage domains of wild-type *Fok*I or the obligate heterodimeric *Fok*I variant KV/EA [55] and the LRGS linker [54]. The target plasmid pCMV.LacZsPK∂GFP was generated by replacing the “31” target site of pCMV.LacZs31∂GFP [59] by the ZFN target site aGTTTGCGCCtaactGAAGGTGACa (capital letters indicate target site for ZFN). The donor plasmid pJet.SAE8586Neo (Fig 1A) consists of (i) a splice acceptor (SA) [60]; (ii) a cDNA consisting of *prkdc* exons 85 and 86, which was PCR amplified from pMEPK7 (kindly provided by Masumi Abe) with primers PRK-F/PRK-R (S2 Table); (iii) an SV40 polyadenylation signal (pA); (iv) a *Neo*R cassette comprise the aminoglycoside phosphotransferase coding sequence flanked by the HSV thymidine kinase promoter and an SV40 pA (kindly provided by Stefan Weger); (v) left and right homology arms, which were PCR amplified from Fib.S gDNA.

### Characterization of ZFN

For expression analysis, ZFNs were expressed in HEK293T for immunoblotting as previously described [59]. The *in vitro* cleavage assay was basically performed as defined before [61]. Briefly, a target DNA was amplified by PCR from Fib.S gDNA using primers IV-F/IV-R (S2 Table). ZFNs were *in vitro* transcribed/translated with the TNT SP6 Coupled Reticulocytes Lysate System (Promega), 150 ng of target DNA was mixed with the reticulocyte lysates, incubated for 1.5 h at 37°C, and analyzed on a 1.5% agarose gel. The plasmid-based gene targeting assay was conducted as described before [59]. Flow cytometry to determine the percentage of EGFP and REX positive cells was performed on FACSCalibur with CellQuestPro software (BD Biosciences).

### Gene targeting in RS-SCID cells

For targeted integration into Fib.S fibroblasts, 1x10^5^ cells were transfected 24 h after seeding with Lipofectamine 2000 (Life Technologies). 1.6 μg of endotoxin-free DNA was mixed with 4.8 μl of transfection reagent in 200 μl OptiMEM (Gibco). The ZFN expression plasmids were co-transfected with the donor pJet.SAE8586Neo at different ratios and filled up with pUC118 to 1.6 μg. Selection with 500 μg/ml of G418 (Sigma-Aldrich) was applied 5 days after transfection for 7 days. iPSCs were grown feeder-free before and after transfection. 3x10^6^ cells were nucleofected with 10 μg of pJET.SAE8586Neo and 5 μg of each ZFN expression plasmid using the Mouse ES Cell Nucleofector Kit (LONZA) and Nucleofector II with program A-030. After 5 days of recovery, G418 selection was applied for 7 days at a concentration of 400 μg/ml. After 1 week, iPSC clones were isolated and cultivated on feeders.

### Genotyping by PCR or Southern blotting

Genomic DNA was extracted with the QIAamp DNA Blood Mini Kit (QIAGEN). G418 selected fibroblast and iPSC clones were analyzed for legitimate targeted integration by inside-out PCR using Phire Hot Start II DNA polymerase kit (Thermo Scientific). RNA was isolated with TRIzol (Life Technologies), and all RT-PCR reactions performed with the QuantiTect Reverse Transcription Kit (QIAGEN). All used primers are listed in S2 Table. For Southern blot analysis [62], genomic DNA was digested with *Eco*RV or *Bam*HI, separated on a 0.8% agarose gel and transferred to Biodyne B nylon membrane (PALL Life Sciences). DNA was hybridized with a ^32^P-labeled fragment of PRE (for detection of the reprogramming vector) or NeoR (for detection of donor copies) using the DecaLabel DNA Labeling Kit (Fermentas). Labeled *Hind*III digested Lambda DNA was used as a marker.

### Functional tests to assay DNA-PK activity

To measure DNA-PK dependent RPA2 phosphorylation, 8x10^5^ fibroblasts were treated with 1 μM of camptothecin (Sigma-Aldrich) for 1 h. Cells were harvested in RIPA buffer supplemented with Complete Protease Inhibitor and PhosSTOP phosphatase inhibitor cocktails (both Roche). Western blot was basically performed as described before [63,64]. RPA2 and ß-actin were detected with rat anti-RPA32 (1:1000, 4E4, Cell Signaling) and rabbit anti-ß-actin (1:1000, Cell Signaling), respectively, and visualized with HRP-conjugated anti-rat and anti-rabbit antibodies (1:20,000, Dianova) and West Pico Chemiluminescence substrate (Thermo Scientific). For the colony survival assay, 1x10^5^ fibroblasts were treated 1 day after seeding with the indicated amounts of bleomycin (Sigma-Aldrich) for 2 h. Cells were washed with PBS, trypsinized and 5,000 cells seeded into a 10-cm plate (N = 3). After 4 days the plates were stained with 0.5% (w/MeOH) crystal violet (Sigma-Aldrich) and colonies counted.

### Generation, excision and characterization of iPSC

Murine adult fibroblasts were extracted from ears of 6-week old NOD/ShiLtJ and NOD.CB17-Prkdc scid/J male mice as described before [62]. Fibroblast from 12-week old NOD.CB17-Prkdc scid/J mouse gave rise to spontaneously transformed Fib.S. The “4-in-1” reprogramming vector pRRL.PPT.SF.mOKSMco.idTom.PRE, co-expressing the transcription factors Oct4, Klf4, Sox2 and c-Myc with the fluorescent marker tdTomato, has been previously described [35]. To generate versions that allow for Flp recombinas-mediated excision (pRRL.PPT.SF.mOKSMco.idTom.PRE.FRT), FRT sites were introduced into the promoter-deprived U3 region. Virus production has been described elsewhere [65]. The reprogramming was conducted as described before [35]. Briefly, NOD.CB17-Prkdc scid/J or NOD/ShiLtJ-derived fibroblasts were seeded in MEF medium on gelatin-coated 6-well-plates at 8x10^4^/well for transduction. After 2 days, cells were transduced with an MOI of 5 and incubated for 8 h, following 2 times washing with PBS. MEF medium with 2 mM VPA (Sigma Aldrich) was added. After 4 days medium was changed to iPS medium with VPA, and after 7 days 3i was added. After 14 days, emerging iPSC colonies were isolated and expanded for characterization. A total of 12 iPSC clones derived from NOD.CB17-Prkdc scid/J (iPS.S) were initially characterized by assessing expression of SSEA-1 by flow cytometry and staining of alkaline phosphatase (Millipore) followed by documentation with the Olympus IX71 system. Determination of the vector copy number (VCN), teratoma formation, Flp recombinase-mediated excision, fluorescence *in situ* hybridization (FISH) and pluripotency factors RT-PCR analysis have been described previously [35,62,66]. Clone iPS.S6 was used for gene targeting and three out of 41 corrected clones (iPS.T8, iPS.T25, iPS.T44) were characterized in detail. The parental uncorrected clone iPS.S6 was included as a negative control, a wild-type NOD/ShiLtJ derived clone (iPS.WT) as a positive control.

### *In vitro* T-cell differentiation

The protocol was adapted from previously published work [48,49]. For embryoid body (EB) formation, iPSCs were split with Collagenase IV (Gibco) and 5x10^4^ cells were cultured in suspension plates in 2 ml of EB medium [IMDM (Biochrom AG) with 15% ES cult FCS (Stem Cell Technologies), 5% PFHM II (Gibco), P/S, L-Glutamine, 50 μg/ml P-VitC, 150 mM MTG, 200 μg/ml human transferrin (Sigma-Aldrich)] in a normoxic incubator on a shaker at 60 rpm. At day 2.5, 0.5 ml of EB medium plus cytokines rhBMP-4, activinA, rhVEGF165 and rhFGF-2 at 5 ng/ml final concentration each (all R&D Systems) was added. At day 8, EBs were harvested, washed with PBS and collected in Trypsin-EDTA, diluted 1:15 in Collagenase IV. After 30 min, 2.5 ml of cell dissociation buffer (Gibco) was added and cells transferred through a 70-μm mesh. Hematopoietic progenitor cells (HPCs) were washed with PBS and analyzed for CD41/cKit expression by flow cytometry prior to hematopoietic expansion. To this end, 10^6^ EB-derived HPCs were cultivated for 3 days under hypoxic conditions in STFV medium [IMDM, 10% OP9-tested FCS, P/S, L-glutamine, 10 ng/ml mSCF, 20 ng/ml mTPO, 100 ng/ml rhFlt3-L (all Peprotech), and 40 ng/ml rhVEGF165 (R&D Systems) (final concentration each)]. At day 3, cells were harvested through a 100-μm mesh and washed with PBS prior to *in vitro* T-cell differentiation. To this end, up to 3x10^5^ expanded HPCs or 0.5-1x10^5^ HSCs were added in T-cell differentiation medium [OP9 medium, supplemented with 1 ng/ml mIL-7 (Peprotech) and 5 ng/ml rhFlt3-L]. After 3 days, 2 ml medium was added and cultivation continued for up to 4 weeks. Every 7 days cells were harvested through a 100-μm mesh, washed with PBS, transferred to a new OP9-DL1 cell layer, and analyzed for T-cell differentiation by flow cytometry.

### Analysis of T-cell phenotype and genotype

For flow cytometric analysis, cells were resuspended in FACS buffer [PBS supplemented with 2% FCS, 1 mM EDTA and 0.1% sodium azide (both Sigma-Aldrich)]. To stain for pluripotency marker SSEA-1, iPSCs were rinsed with PBS and stained with biotinylated anti-SSEA-1 antibody (eBioscience) diluted in FACS buffer for 20 min at 4°C. After rinsing the secondary staining was performed with a streptavidin-APC antibody (eBioscience). Hematopoietic cells were pretreated with Mouse BD Fc block (BD Biosciences) before antibody staining. Antibody staining was performed for 20 min at 4°C. EB-derived HPCs were stained with CD41-PE, cKit-APC, or respective isotype controls (all eBioscience). iPSC-derived T-cells were stained with CD44-PE and CD25-APC, or CD4-PE and CD8-APC. Viability staining with 7-AAD was performed for 2 min during the last rinsing, before samples were measured on a FACSCalibur. Alternatively, iPSC-derived T-cells were stained with CD45-APC-Cy7, CD4-PerCPR-Cy5.5, CD8-PE-Cy7 (all BD Biosciences), CD44-PE, CD25-APC, TCRß-FITC (eBioscience) and DAPI, before analysis on a FACSCanto II with FACSDiva (BD Biosciences). All samples were analyzed with FlowJo software (Tree Star). T-cell receptor diversity was analyzed by CDR3 spectratyping as previously described [67].

### Statistics

All experiments were performed at least three times. Error bars represent standard deviation (SD). Statistical significance was determined with a two-sided Student's *t*-test with unequal variance.

### Accession numbers

The National Center for Biotechnology Information (NCBI) Nucleotide database (http://www.ncbi.nlm.nih.gov/nuccore) accession number for the ZFN target site in intron 84 of the *prkdc* gene on mouse chromosome 16 is AB030754: 189732.

## Supporting Information

S1 Table Off-target site analysis. (DOCX)

S2 Table Sequences of primers used for characterization of gene targeted cells. (DOCX)

S1 Text Supplementary Methods. (DOCX)

S1 Fig ZFNs targeting prkdc locus. (A) Schematic view of ZFN binding site (ZFN BS) in *prkdc*. The position and sequence of the ZFN BS in intron 84 is shown. F1, F2, F3 indicate target triplets for each binding half-sites. The spacer is highlighted in italics, mut* indicates the position of the SCID mutation. (B) Sequence of *prkdc*-specific ZF modules. Amino acid sequences of the ZF modules that recognize F1, F2, or F3 target triplets for the left and right target half-sites (5’ to 3’ orientation). Three ZFs for the left binding half-site (L1, L2, L3) and two for the right target half-site (R1, R2) have been selected and tested for their ability to activate a beta-galactosidase (ßgal) reporter **[32].** (C) Expression analysis of ZFNs. ZFN-encoding plasmids were transfected in 293T cells and protein levels detected by immunoblotting using Odyssey IRDye antibodies. L1, L2, L3, ZFN left subunits 1, 2 or 3 with WT *Fok*I domain; L1H, L2H, L3H, ZFN left subunits 1, 2 or 3 with “EA” obligate heterodimeric *Fok*I domain **[55];** L2N, ZFN left subunit L2 with “EA” obligate heterodimeric *Fok*I domain without NLS signal; R1, R2, ZFN right subunits 1 or 2 with WT *Fok*I domain; R1H, R2H, ZFN right subunits 1 or 2 with “QK” obligate heterodimeric *Fok*I domain **[55];** R1N, R2N, ZFN right subunits 1 or 2 with “QK” obligate heterodimeric *Fok*I domain without NLS signal. GFP served as transfection and loading control. (D) *In vitro* cleavage assay. ZFN pair L2N and R1N was *in vitro* transcribed/translated and mixed with a ZFN BS-containing PCR product. Cleavage reaction **[61]** was analyzed on a 1.5% agarose gel. Size markers are indicated. (E) Plasmid-based gene correction assay. 293T cells were transfected with target plasmid, repair matrix and ZFN or ***Sce***I plasmids in order to induce episomal homology-directed repair **[59].** Y axis shows the percentage of relative gene correction frequency, which is calculated as GFP-positive cells (target plasmid corrected) REX-positive cells (transfected cells). (+), positive control *Sce*I; (-), negative control *Sce*I∆; WT, L2 + R1 with homodimeric *Fok*I domain; OH, L2 + R1 with obligate heterodimeric *Fok*I domain;-NLS, L2 + R1 with OH *Fok*I domain without NLS signal.
(TIF)

S2 Fig Cellular characterization of iPSC clones. (**A**) Ssea-1 expression. Surface expression of pluripotency marker Ssea-1 was measured by flow cytometry. Fib.S, NOD.SCID-derived ear fibroblasts; ES.CCE, murine ES cell line; iPS.S1, iPS.S6 and iPS.S12, NOD.SCID-derived iPSC clones. (**B**) Alkaline phosphatase staining. NOD.SCID-derived iPSCs were stained using the Alkaline Phosphatase Detection Kit and analyzed by microscopy. (**C**) Spectral karyotyping (SKY). Multicolor fluorescent *in situ* hybridization (FISH) based karyotyping was used to assess genome integrity. iPS.T8 and iPS.T44, gene targeted iPSC clones. (**D**) Teratoma Assay. Hematoxylin/eosin-stained sections of teratoma isolated 8 weeks after injection of iPSCs into NSG mice. Detection of ectodermal, endodermal and mesodermal tissues. iPS.T8 and iPS.T44, gene targeted iPSC clones.
(TIF)

S3 Fig Molecular characterization of iPSC clones. (**A**) Excision of the reprogramming cassette. iPSCs have been treated with Flp recombinase to remove the lentiviral reprogramming cassette. Excision was confirmed by detection of the viral PRE elements via Southern blot. Genomic DNA was digested with *Eco*RV or *Bam*HI. Positions of restriction sites in reprogramming cassette and the expected minimal band sizes are indicated on top. M, DNA size marker; S6 and S6X, SCID iPSC clones; T25 and T25X, targeted iPSC clones; WT and WTX, wild-type iPSC clones; X indicates clones with excised reprogramming cassette. (**B**) Random integration of donor. The *NeoR* cassette, as an indicator of donor DNA, was detected by Southern blot to confirm targeted integration in *prkdc* intron 84. Genomic DNA was digested with *Eco*RV or *Nde*I. Positions of restriction sites in the modified intron 84 and the expected band sizes are indicated on top. (**C**) Determination of vector copy number (VCN). Copy number of the lentiviral reprogramming vector was assessed by quantitative PCR [62], and is indicated as PRE per endogenous Flk3 copy. Fib, murine ear fibroblasts; iPS.1X and iPS.2X, iPSC clones with excised reprogramming cassette; iPS.S1, iPS.S2, iPS.S3, iPS.S4, iPS.S6, iPS.S8, iPS.S9 and iPS.S12, NOD.SCID-derived iPSC clones.
(TIF)

S4 Fig Control of *in vitro* differentiation. Samples were stained with CD41-PE, cKit-APC or their corresponding isotype controls, and 7-AAD after dissociation of 8 d matured embryoid bodies (EBs). All plots were pre-gated on FSC/SSC and 7-AAD-negativity. Red numbers indicate percentage of cells in each quadrant. iPS.WT and iPS.WT X, wild-type iPSC clones; iPS.S6 and iPS.S6 X, SCID iPSC clones; iPS.T25, iPS.T25X and iPS.T44, targeted iPSC clones; X indicates iPSC clones with excised reprogramming cassette.
(TIF)

S5 Fig Analysis of T cell receptor diversity of *in vitro* generated T cells by spectratyping. Quantitative PCR was performed on genomic DNA isolated from *in vitro* generated T cells. Exemplarily shown are PCR analyses of the variable beta chains Vß1, Vß6, Vß8.1, Vß8.3, Vß10, Vß12, Vß14 and Vß20. X axis indicates PCR fragment size in bp, Y axis shows quantity of PCR amplicons. Thymus, control DNA of cells isolated from thymus; HSC, *in vitro* generated T cells; iPS.WTX, wild-type iPSC clone; iPS.S6 and iPS.S6X, SCID iPSC clones; iPS.T25, iPS.T25X and iPS.T44, targeted iPSC clones; X indicates clones with excised reprogramming cassette.
(TIF)
