# Supplementary material for: Rescue of DNA-PK Signaling and T-Cell Differentiation by Targeted Genome Editing in a prkdc Deficient iPSC Disease Model
Source: PLoS Genet. 2015 May 22;11(5):e1005239. doi: 10.1371/journal.pgen.1005239 (PMC4441453; doi:10.1371/journal.pgen.1005239)
Supplement: S1 Table — (DOCX) [file pgen.1005239.s001.docx]

**S1 Table. Off-target site analysis.**

| **ID** | **PROGNOS ranking^1^** | **Nucleotide differences** | | | **Match Type** | **Closest gene** | **Chromo-some** | **Genomic region** | **Target (5'-3')^2^** | **iPSC clone sequence** | | **Primer sequences (5'-3')** | | |
| --- | --- | --- | --- | --- | --- | --- | --- | --- | --- | --- | --- | --- | --- | --- |
|  |  | **Left** | **Right** | **Total** |  |  |  |  |  | **T25** | **T44** | **forward** | **reverse** |  |
| Target | 100 | 0 | 0 | 0 | L05R | *Prkdc* | 16 | Intron | GTTTAGTTTGCGCCTAACTGGAGGTGACAGTAT | wt^3^ | wt^3^ | TGCATGCACTGAGGGCTTTCC | GAGGACAGCCTTGGCTATGTG |  |
| OT_01 | 88,12 | 0 | 1 | 1 | R05R | *Iqsec1* | 6 | Intron | TCAAAGTCACCTCCAATCTGGAGGTG**G**CTCCAG | wt | wt | ATCCCACATGGAACCTGACTGGC | CATGTGAATGCCATCTCCCCAAC |  |
| OT_02 | 84,53 | 0 | 1 | 1 | R05R | *Slc39a9* | 12 | Exon | AAAATGTCACCTCCTGGTGGGAG**T**TGACACAGT | wt | wt | TCCTGGTGATGTCAGTCCTGTG | CTGCTGGCTGACCTAGTGAC |  |
| OT_03 | 82,05 | 0 | 1 | 1 | R05R | *Eepd1* | 9 | Intron | ACAAAGTCACCT**T**CCGGTAGGAGGTGACATGGG | n.d. | | CTGACACACAGGCATAGACACAGA | CCTCTTTGTCAAGCAGCTCTTGCA |  |
| OT_04 | 79,72 | 1 | 0 | 1 | L05R | *Cd5* | 19 | Intron | ATCAGGTTTG**T**GCCTCCCTGGAGGTGACCAAAT | wt | wt | CAAGGCTCATGAGTTGGGGAC | AGGCTGAATACCTGTGCATACCG |  |
| OT_05 | 74,87 | 1 | 1 | 2 | R06L | *Gm20554* | 13 | Exon | GCTAGG**C**CACCTCCTGGAGGGGCGCAAA**A**CCCTG | wt | wt | CAAGACCAAGAGTAAGCAAGCCG | CGCTGTTCTCACCCTTGTCTG |  |
| OT_06 | 74,63 | 2 | 0 | 2 | L05R | *Pag1* | 3 | Intron | ATACGGT**G**T**A**CGCCTCCGTGGAGGTGACTGTTT | wt | wt | GTCAGCTGCGGTGCTCTTGAA | GACCTGAGAGGCAAGAGCAGA |  |
| OT_07 | 74,18 | 1 | 1 | 2 | R05L | *Cpne4* | 9 | Intron | CTGAGGTC**T**CCTCCAGCCTGGCGtAAA**G**CAACA^4^ | wt | wt | AGGACTTGTGACAGGTGCACCC | AAAGTGGGCCTTTGGCGTCC |  |
| OT_08 | 74,18 | 1 | 1 | 2 | R06L | *Pax6os1* | 2 | Intron | GTTTAGTC**C**CCTCCCCCAAAGGCGCAAA**T**ATCTA | wt | wt | CCTGACATGATGTGCTGAAACCC | CTGTATTCCTTTGTCCTGCAGTCC |  |
| OT_09 | 72,71 | 2 | 0 | 2 | R05L | *Flnc* | 6 | Intron | CATTGGTCACCTCCTTCTAGG**G**GCAA**T**CTACAC | wt | wt | CTCTAGCCCCACCCCACATT | CCTCTCTCAGCTCAGTGCTGT |  |
| OT_10 | 72,71 | 2 | 0 | 2 | L06R | *Fam189a1* | 7 | Intron | CCTAC**C**TTTGC**C**CCACCAGAGGAGGTGACGTAGT | wt | wt | TGTTTGGGGATGGGGGACAG | TGTGAGTGTGGGAAACAGTGGG |  |
| OT_11 | 72,71 | 2 | 0 | 2 | R06L | *Prkg2* | 5 | Intron | TCCCAGTCACCTCCAGCCCAGG**A**GCA**G**ACCAGCT | wt | wt | CTCAACAGTCTCTAACTTCGTACCAAACC | TGCTTGGGTTCCCAGGGAATG |  |
| OT_12 | 72,40 | 0 | 2 | 2 | R05L | *Sema5a* | 15 | Intron | CTAGTGT**TG**CCTCCAACTCGGCGCAAACTGAAC | wt | wt | CTCTGTCACTATGGCTCCATCAAC | CCCACTACCACTGCTGTATAGTGT |  |
| OT_13 | 72,13 | 0 | 2 | 2 | R06L | *Fam105a* | 15 | Intron | TGTGGG**C**CAC**A**TCCTCTCAAGGCGCAAACCCCGG | wt | wt | CGGTGTCCAACAACCCCATTTAC | AGAGCGAAGGGTGCTTGTGG |  |
| OT_14 | 71,14 | 1 | 1 | 2 | R05L | *Nek10* | 14 | Intron | ATCATGTC**T**CCTCCACAAAGG**G**GCAAACTTTCT | wt | wt | GTGTGTGACTGCACTTGGCAATG | CCCAACCATTCCTGAGTAACAGC |  |
| OT_15 | 70,99 | 0 | 2 | 2 | L05R | *Asb18* | 1 | Intron | CTGATGTTTGCGCCCAAAT**TT**AGGTGACACAGA | wt | wt | GCTGCTTTCAGGTGATCCATGTG | TGCTTGTGGACTCCAGGAAGG |  |

^1^ Calculated with ZFN v.2.0 ranking system (http://baolab.bme.gatech.edu/cgi-bin/prognos/prognos.cgi). The top 15 sites were chosen as described in Supplementary Methods.

^2^ ZFN target site in *prkdc* and predicted off-target sites are underlined, differences to the target site are highlighted in bold letters.

^3^ The sequence of the target site in the two iPSC clones refers to the unmodified allele.

^4^ The C/T SNP rs50495895 in the ZFN off-target site was found to be a T (indicated as lower case t) in both of our sequenced iPSC clones.

n.d., not determined because of PCR failure.
