# Supplementary material for: Rescue of DNA-PK Signaling and T-Cell Differentiation by Targeted Genome Editing in a prkdc Deficient iPSC Disease Model
Source: PLoS Genet. 2015 May 22;11(5):e1005239. doi: 10.1371/journal.pgen.1005239 (PMC4441453; doi:10.1371/journal.pgen.1005239)
Supplement: S2 Table — (DOCX) [file pgen.1005239.s002.docx]

## S2 Table. Sequences of primers used for characterization of gene targeted cells.

| primer | 5´→ 3´ | application |
| --- | --- | --- |
| AD-F | gaggggtactacttactggcttgtt | allelic discrimination PCR |
| AD-R | cagctctctagctttctagtcagaca |  |
| J5-F | cctgtaagtcatgggaaacaca | 5’-junction PCR to assess targeted integration |
| J5-R | GCCTTCGACTTGGGTGGAAACATTC |  |
| J3-F | cgtgatattgctgaagagcttg | 3’-junction PCR to assess targeted integration |
| J3-R | ctcagcattgtctgctcaaaac |  |
| RT-F | catgcgtttggatcagctac | transgene expression by RT-PCR |
| RT-R | cggccaagcttaggaggt |  |
| IV-F | aagaaacgggtctcatgtgc | PCR target for *in vitro* cleavage assay |
| IV-R | aggttcggaggaggattgtt |  |
| PRK-F | GTTTTGAGCAGACAATGCTGAGAAAAGGAG | donor cloning of exons 85/86 |
| PRK-R | AGCATATTTGTCATATTCTTTATTTTTTACATGTGTGGAGGTCA |  |
