# Supplementary material for: Rescue of DNA-PK Signaling and T-Cell Differentiation by Targeted Genome Editing in a prkdc Deficient iPSC Disease Model
Source: PLoS Genet. 2015 May 22;11(5):e1005239. doi: 10.1371/journal.pgen.1005239 (PMC4441453; doi:10.1371/journal.pgen.1005239)
Supplement: S1 Text — (DOCX) [file pgen.1005239.s003.docx]

**S1 Text. Supplementary Methods**

**Off-target site analysis**

The identification of potential off-target cleavage sites for the *prkdc*-specific ZFNs used in this study was performed using the bioinformatics tool PROGNOS [68]. The software (http://baolab.bme.gatech.edu/cgi-bin/prognos/prognos.cgi) provides a ranked list of potential off-target sites. The 15 sites listed in Supplementary Table 1 were chosen using the following criteria: (i) homodimeric sites in introns or exons with up to 1 mismatch compared to the target sequence; (ii) heterodimeric sites in introns or exons with up to 2 mismatch compared to the target sequence; (iii) ZF score threshold above 70. The 15 predicted off-target sites were PCR amplified using genomic DNA isolated from the two iPSC clones T25 and T44 as a template and the primer pairs suggested by PROGNOS (listed in Supplementary Table 1). The PCR amplicons were analyzed by Sanger sequencing (GATC Biotech, Constance, Germany) and compared to the wild-type sequences deposited in an online database (http://genome-euro.ucsc.edu/cgi-bin/hgGateway).

**Supplementary Reference**

68. Fine EJ, Cradick TJ, Zhao CL, Lin Y, Bao G (2013) An online bioinformatics tool predicts zinc finger and TALE nuclease off-target cleavage. Nucleic Acids Res 42: e42.
