# Supplementary figures and images for: Rescue of DNA-PK Signaling and T-Cell Differentiation by Targeted Genome Editing in a prkdc Deficient iPSC Disease Model
Source: PLoS Genet. 2015 May 22;11(5):e1005239. doi: 10.1371/journal.pgen.1005239 (PMC4441453; doi:10.1371/journal.pgen.1005239)

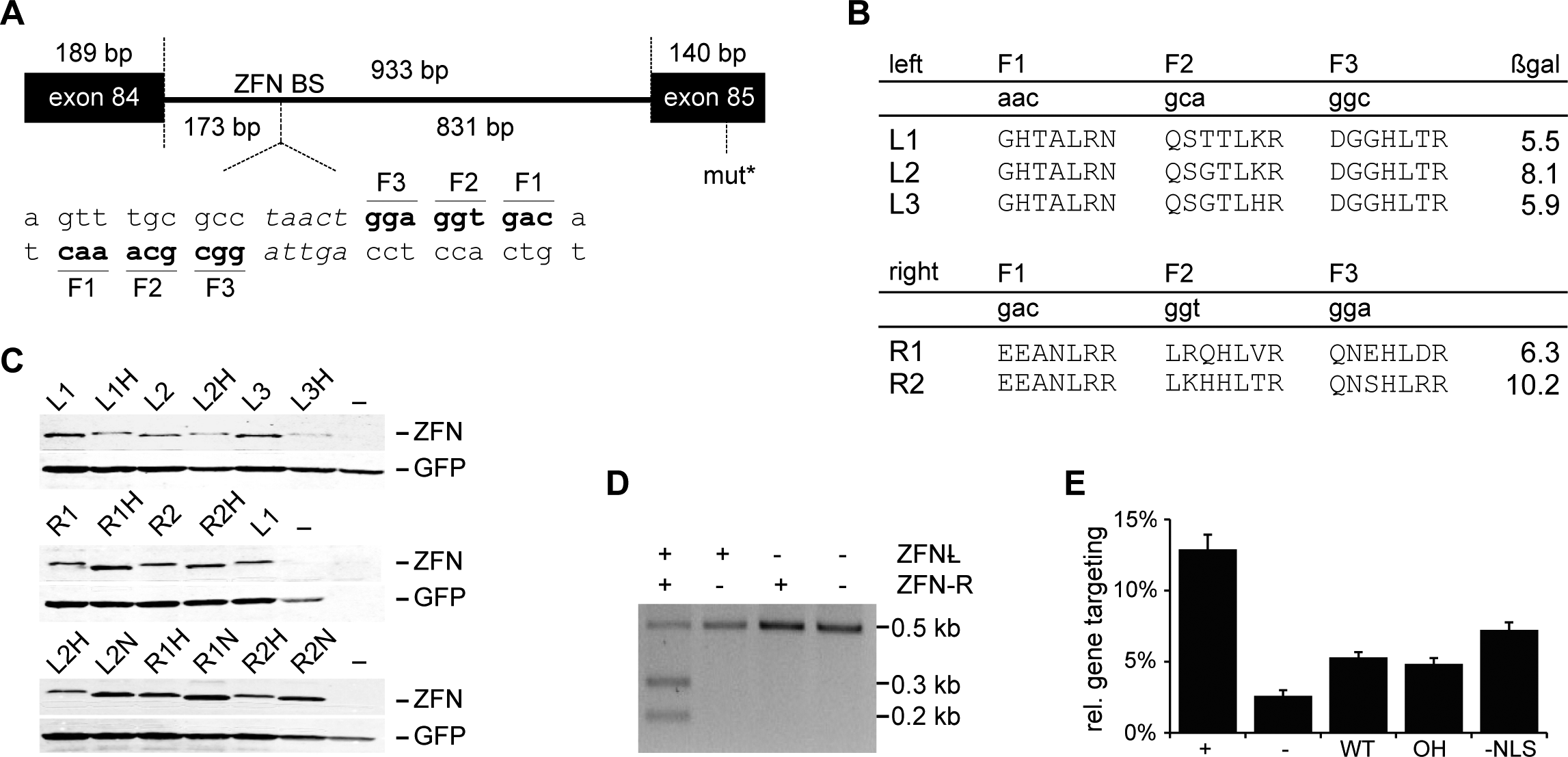

Supplement: S1 Fig — (A) Schematic view of ZFN binding site (ZFN BS) in prkdc. The position and sequence of the ZFN BS in intron 84 is shown. F1, F2, F3 indicate target triplets for each binding half-sites. The spacer is highlighted in italics, mut* indicates the position of the SCID mutation. (B) Sequence of prkdc-specific ZF modules. Amino acid sequences of the ZF modules that recognize F1, F2, or F3 target triplets for the left and right target half-sites (5’ to 3’ orientation). Three ZFs for the left binding half-site (L1, L2, L3) and two for the right target half-site (R1, R2) have been selected and tested for their ability to activate a beta-galactosidase (ßgal) reporter [32]. (C) Expression analysis of ZFNs. ZFN-encoding plasmids were transfected in 293T cells and protein levels detected by immunoblotting using Odyssey IRDye antibodies. L1, L2, L3, ZFN left subunits 1, 2 or 3 with WT FokI domain; L1H, L2H, L3H, ZFN left subunits 1, 2 or 3 with “EA” obligate heterodimeric FokI domain [55]; L2N, ZFN left subunit L2 with “EA” obligate heterodimeric FokI domain without NLS signal; R1, R2, ZFN right subunits 1 or 2 with WT FokI domain; R1H, R2H, ZFN right subunits 1 or 2 with “QK” obligate heterodimeric FokI domain [55]; R1N, R2N, ZFN right subunits 1 or 2 with “QK” obligate heterodimeric FokI domain without NLS signal. GFP served as transfection and loading control. (D) In vitro cleavage assay. ZFN pair L2N and R1N was in vitro transcribed/translated and mixed with a ZFN BS-containing PCR product. Cleavage reaction [61] was analyzed on a 1.5% agarose gel. Size markers are indicated. (E) Plasmid-based gene correction assay. 293T cells were transfected with target plasmid, repair matrix and ZFN or SceI plasmids in order to induce episomal homology-directed repair [59]. Y axis shows the percentage of relative gene correction frequency, which is calculated as GFP-positive cells (target plasmid corrected) REX-positive cells (transfected cells). (+), positive control SceI; (-), negative co [file pgen.1005239.s004.tif]

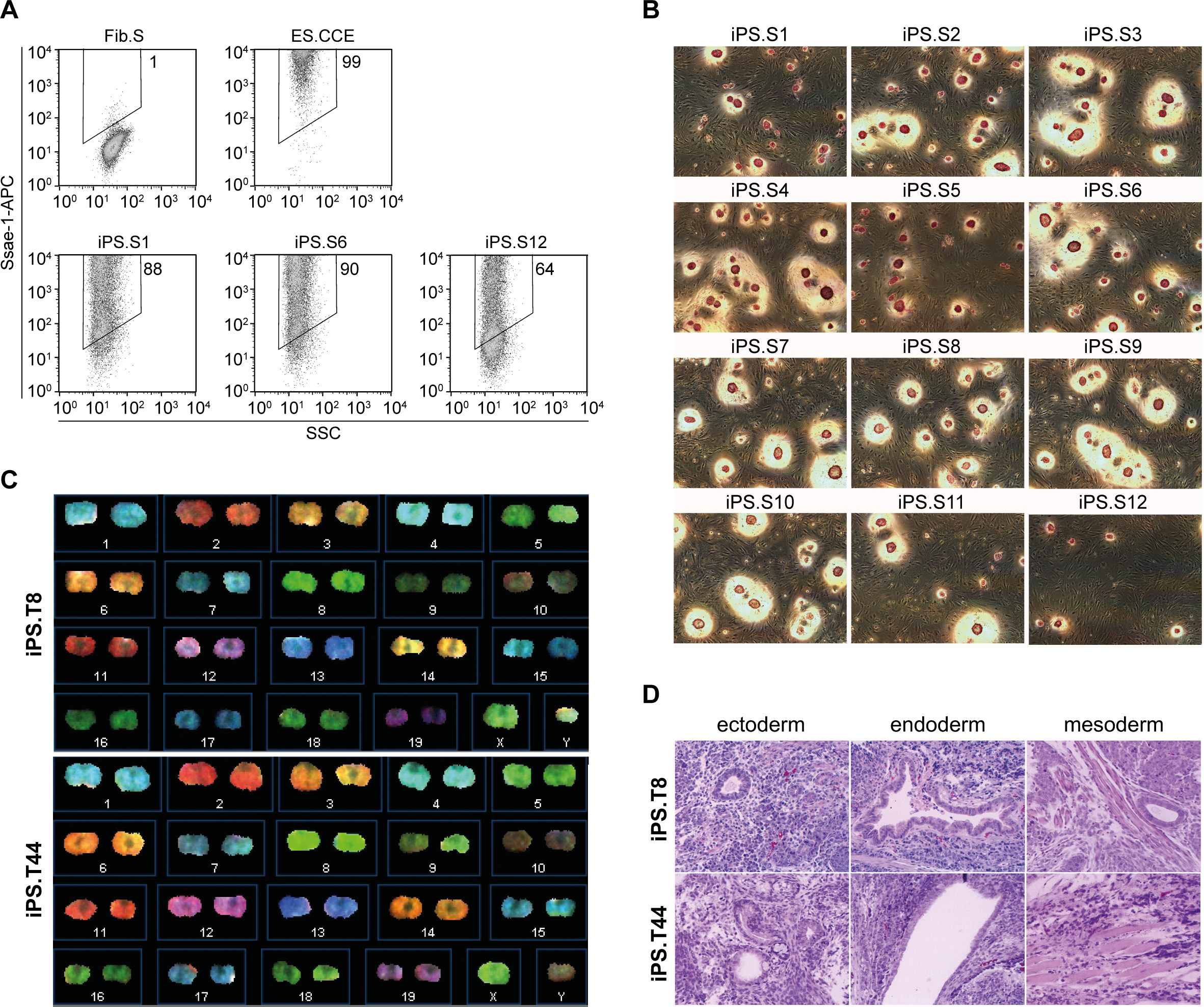

Supplement: S2 Fig — (A) Ssea-1 expression. Surface expression of pluripotency marker Ssea-1 was measured by flow cytometry. Fib.S, NOD.SCID-derived ear fibroblasts; ES.CCE, murine ES cell line; iPS.S1, iPS.S6 and iPS.S12, NOD.SCID-derived iPSC clones. (B) Alkaline phosphatase staining. NOD.SCID-derived iPSCs were stained using the Alkaline Phosphatase Detection Kit and analyzed by microscopy. (C) Spectral karyotyping (SKY). Multicolor fluorescent in situ hybridization (FISH) based karyotyping was used to assess genome integrity. iPS.T8 and iPS.T44, gene targeted iPSC clones. (D) Teratoma Assay. Hematoxylin/eosin-stained sections of teratoma isolated 8 weeks after injection of iPSCs into NSG mice. Detection of ectodermal, endodermal and mesodermal tissues. iPS.T8 and iPS.T44, gene targeted iPSC clones. (TIF) [file pgen.1005239.s005.tif]

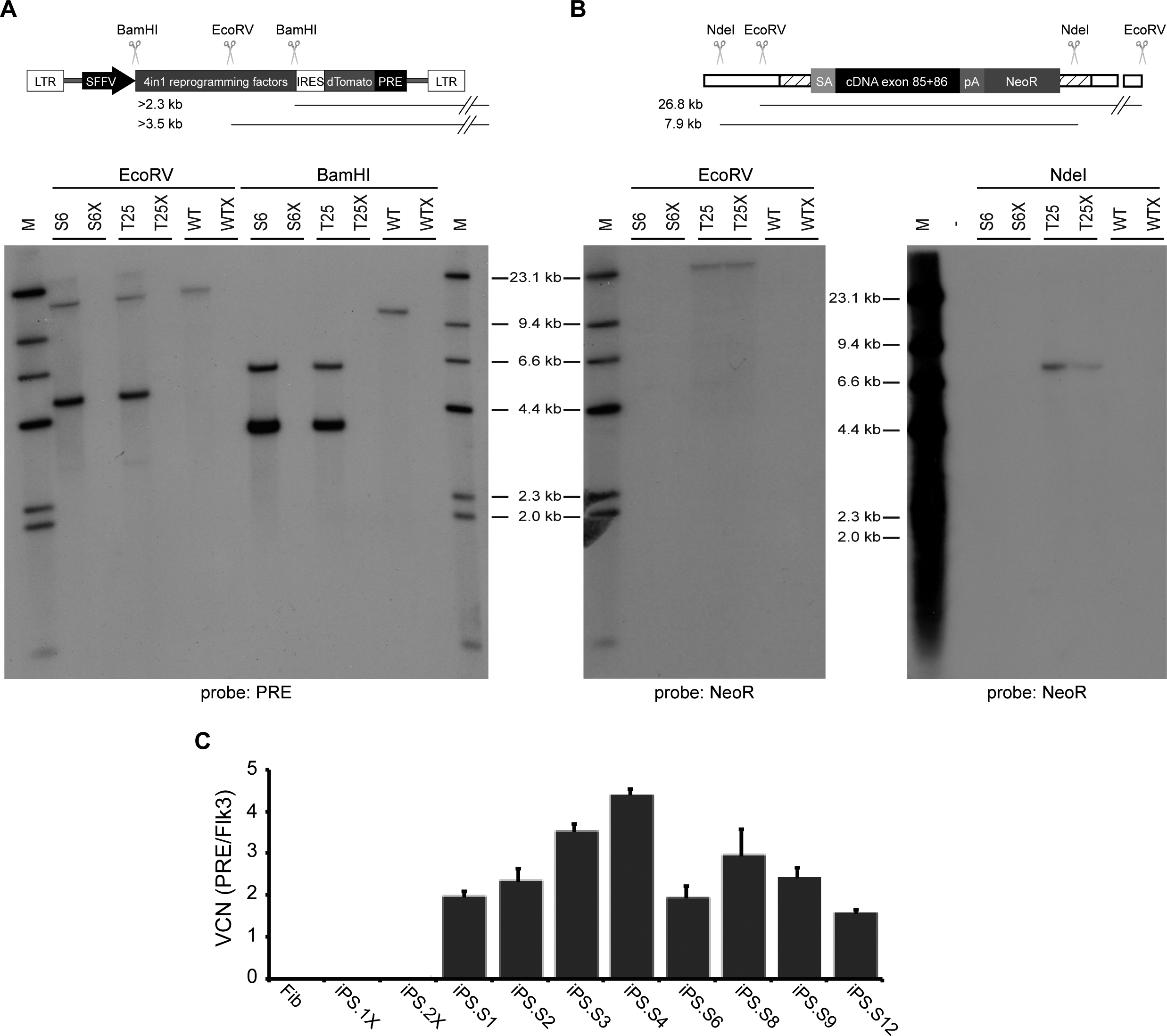

Supplement: S3 Fig — (A) Excision of the reprogramming cassette. iPSCs have been treated with Flp recombinase to remove the lentiviral reprogramming cassette. Excision was confirmed by detection of the viral PRE elements via Southern blot. Genomic DNA was digested with EcoRV or BamHI. Positions of restriction sites in reprogramming cassette and the expected minimal band sizes are indicated on top. M, DNA size marker; S6 and S6X, SCID iPSC clones; T25 and T25X, targeted iPSC clones; WT and WTX, wild-type iPSC clones; X indicates clones with excised reprogramming cassette. (B) Random integration of donor. The NeoR cassette, as an indicator of donor DNA, was detected by Southern blot to confirm targeted integration in prkdc intron 84. Genomic DNA was digested with EcoRV or NdeI. Positions of restriction sites in the modified intron 84 and the expected band sizes are indicated on top. (C) Determination of vector copy number (VCN). Copy number of the lentiviral reprogramming vector was assessed by quantitative PCR [62], and is indicated as PRE per endogenous Flk3 copy. Fib, murine ear fibroblasts; iPS.1X and iPS.2X, iPSC clones with excised reprogramming cassette; iPS.S1, iPS.S2, iPS.S3, iPS.S4, iPS.S6, iPS.S8, iPS.S9 and iPS.S12, NOD.SCID-derived iPSC clones. (TIF) [file pgen.1005239.s006.tif]

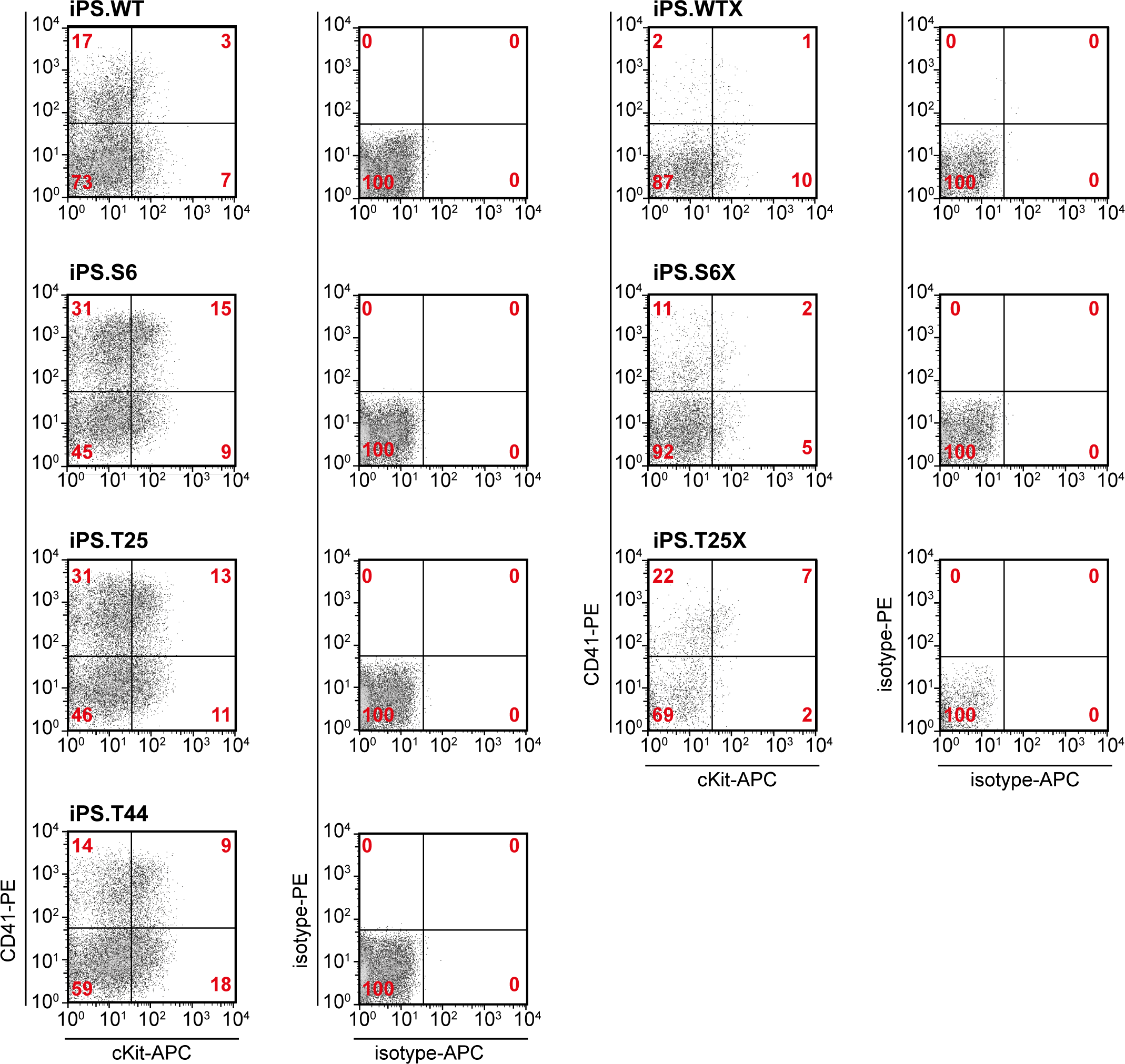

Supplement: S4 Fig — Samples were stained with CD41-PE, cKit-APC or their corresponding isotype controls, and 7-AAD after dissociation of 8 d matured embryoid bodies (EBs). All plots were pre-gated on FSC/SSC and 7-AAD-negativity. Red numbers indicate percentage of cells in each quadrant. iPS.WT and iPS.WT X, wild-type iPSC clones; iPS.S6 and iPS.S6 X, SCID iPSC clones; iPS.T25, iPS.T25X and iPS.T44, targeted iPSC clones; X indicates iPSC clones with excised reprogramming cassette. (TIF) [file pgen.1005239.s007.tif]

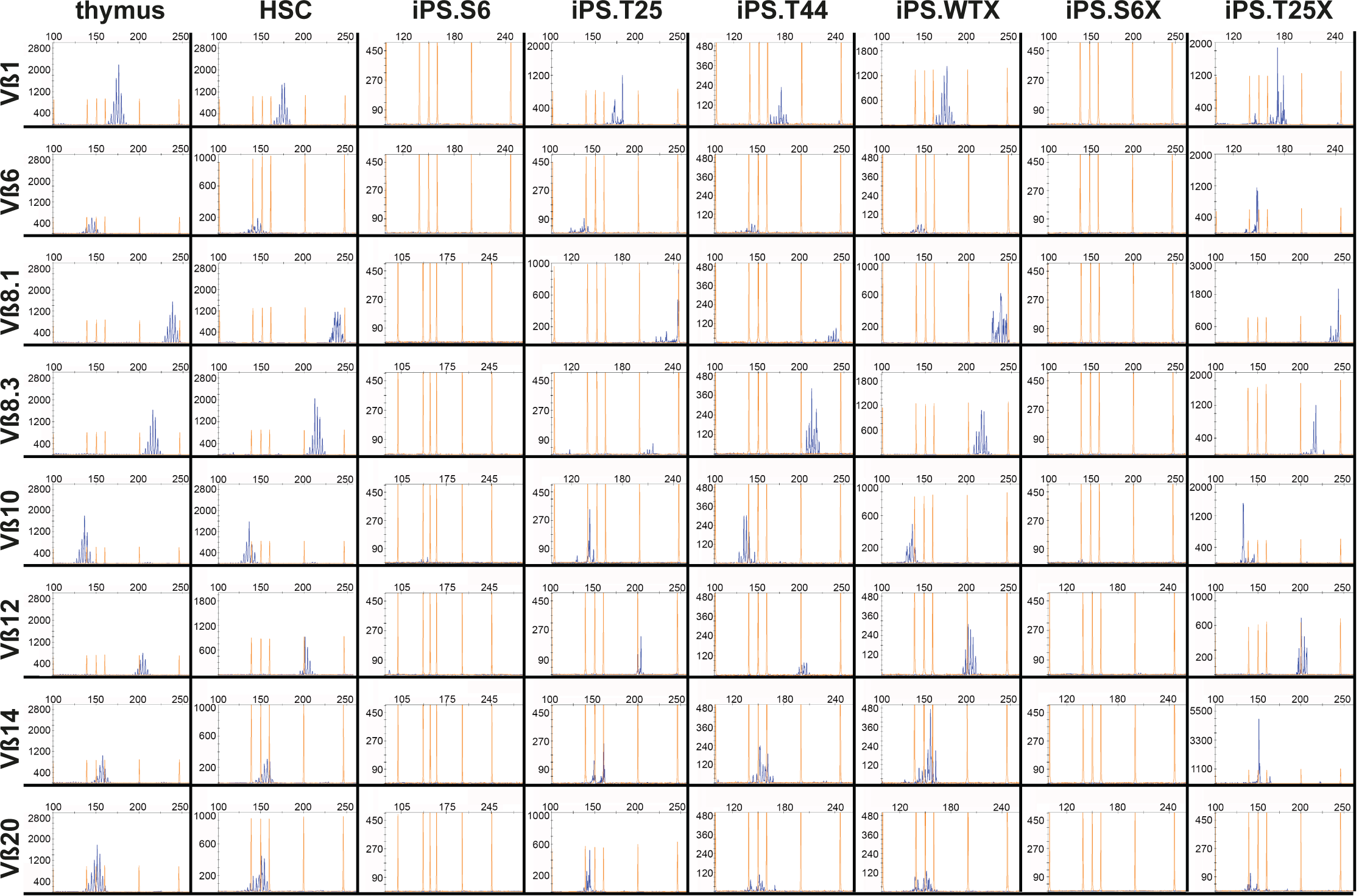

Supplement: S5 Fig — Quantitative PCR was performed on genomic DNA isolated from in vitro generated T cells. Exemplarily shown are PCR analyses of the variable beta chains Vß1, Vß6, Vß8.1, Vß8.3, Vß10, Vß12, Vß14 and Vß20. X axis indicates PCR fragment size in bp, Y axis shows quantity of PCR amplicons. Thymus, control DNA of cells isolated from thymus; HSC, in vitro generated T cells; iPS.WTX, wild-type iPSC clone; iPS.S6 and iPS.S6X, SCID iPSC clones; iPS.T25, iPS.T25X and iPS.T44, targeted iPSC clones; X indicates clones with excised reprogramming cassette. (TIF) [file pgen.1005239.s008.tif]
